# 840. Exploring Patient Experiences of Decision-making Around Skin Graft Surgery Using an AI Burn Assessment Tool

**DOI:** 10.1093/jbcr/irag033.279

**Published:** 2026-04-06

**Authors:** Zoe M Gotts, Elizabeth Orme, Emma L Hodgkinson

**Affiliations:** Newcastle Upon Tyne Hospitals NHS Trust, Newcastle Upon Tyne, England; Newcastle Upon Tyne Hospitals NHS Trust, Newcastle upon Tyne, England; Newcastle Upon Tyne Hospitals NHS Trust, Newcastle Upon Tyne, England

## Abstract

**Introduction:**

Artificial intelligence (AI) is increasingly used in burn care to assist clinical decision-making. An AI wound diagnostic device helps assess burn depth and predict healing potential, but there is limited research on how the use of such technology may impact patients surgical decision-making. This evaluation explored patient experiences of decision-making around skin graft surgery where AI-supported assessment was used.

**Methods:**

10 adult inpatients at a regional burns centre who received an AI wound diagnostic scan were invited to complete a questionnaire and participate in a brief semi-structured interview. 5 of the patients went on to have surgery. Quantitative data assessed understanding, confidence, and concerns around surgery and AI. Qualitative data were analysed using reflexive thematic analysis.

**Results:**

Patients demonstrated limited understanding of AI, limited awareness of the role of AI in surgical decision-making and some patients expressed reservations regarding its use. The surgeon's recommendation around the need for surgery was identified as the most important factor in the patients' decision making. Healing time, pain, and long-term recovery were also identified as important factors influencing surgical choices for patients. Importantly, patients had mixed views regarding the impact AI may have on their decision. The relationship between the surgeon and the AI device was a central theme, and patients emphasized the need for transparency regarding the extent to which AI informed the surgeon’s recommendation.

**Conclusions:**

Initial findings suggest that integrating AI into clinical pathways prompts varied emotional and cognitive responses, with implications for consent and confidence. Patient perspectives offer valuable insight into how technology shapes decision-making and can inform best practice in discussing the use of such technology with patients.

**Applicability of Research to Practice:**

This evaluation will inform safe, patient-centred implementation of AI in burn surgery pathways. Findings may guide clinician communication strategies and shared decision-making frameworks.

**Funding for the study:**

This project received no external funding and was registered as a service evaluation with Newcastle Hospitals NHS Foundation Trust.

